# Meeting abstracts from the 7th Danish Emergency Medicine Conference: Copenhagen, Denmark. 24 - 25 November 2016

**DOI:** 10.1186/s13049-017-0364-2

**Published:** 2017-03-17

**Authors:** Osama Bin Abdullah, Johannes Grand, Astha Sijapati, Petrine Nimskov, Finn Erland Nielsen, Jens Christian Schmidt, Noel Pérez, Tanja Kirkegaard, Marianne Fløjstrup, Mikkel Brabrand, Mathias Galthen-Sørensen, Rasa Ramoskiene, Arman Arshad, Annmarie Lassen, Lars Stubbe Teglbjærg, O. Andersen, L. Mørch Jørgensen, D. M. Sivertsen, J. W. Kirk, J. Petersen, H. H. Klausen, A. C. Bodilsen, J. Petersen, T. Bandholm, T. Haupt, D. M. Sivertsen, O. Andersen, Camilla Schade Hansen, Anton Pottegård, Ulf Ekelund, Jakob Lundager Forberg, Helene Kildegaard Jensen, Annmarie Touborg Lassen, Janni Lynggård Bo Madsen, Ole Graumann, Stefan Posth, Pia Iben Pietersen, Lars Konge, Christian B. Laursen, Janni Lynggård Bo Madsen, Søren Nygaard Hansen, Kristian Møller Jensen, Mikkel Brabrand, Rasmus Bo Hasselbalch, Mia Pries-Heje, Lisbet Ravn, Morten Lind, Lars S. Rasmussen, Birgitte Nybo Jensen, Ulrik Havshøj, Daniel Pilsgaard Henriksen, Mikkel Brabrand, Annmarie Touborg Lassen, Hanne H Nygaard, Christian Maschmann, Helene Skjøt-Arkil, Christian Backer Mogensen, Lotte Høeg Hansen, Lena Wittenhoff, Christian Backer Mogensen, Helene Skjøt-Arkil, Iben Duvald, Iben Duvald, Line Jee Hartmann Rasmussen, Steen Ladelund, Thomas Huneck Haupt, Gertrude Ellekilde, Jesper Eugen-Olsen, Ove Andersen, Martin Betzer, Rasmus Lyngby, Mette Elkjær, Christian Jørgensen, Mikkel Brabrand, Bibi Gram, Mia M. Pries-Heje, Rasmus B. Hasselbalch, Lisbet Ravn, Morten N. Lind, Thomas Boel, Peter Sommer Ulriksen, Nadia Hejgaard Jensen, Kristian Møller Jensen, Elise Mølleskov, Iben Østergaard Fog, Mette Rahbek Kristensen, Ellen Jensen

**Affiliations:** 1 Department of Emergency Medicine, Slagelse Hospital, Slagelse, Denmark; 2 Department of Clinical Research and Institute of Regional Health Services Research, University of Southern Denmark, Odense M, Denmark; 3 Department of Anaesthesiology and Intensive Care Medicine, Hospitalsenheden Vest, Herning, Denmark; 4 Emergency Department, Regionshospitalet Herning, Herning, Denmark; 5 University of Aarhus, Arbejdsmedicinsk Klinik, Regionshospitalet Herning, Herning, Denmark; 6 Department of Anaesthesiology, Vejle Sygehus, Vejle, Denmark; 7 Medical Admission Unit 272, Hospital of South West Jutland, Esbjerg, Denmark; 8 Emergency Department, Odense University Hospital, Odense, Denmark; 9 Institute of Regional Health, University of Southern Denmark, Esbjerg, Denmark; 10 Department of Internal Medicine, Odense University Hospital, Svendborg, Denmark; 11 Department of Emergency, Odense University Hospital, Odense, Denmark; 12 Optimed, Clinical Research Centre, Copenhagen University Hospital, Hvidovre, Denmark; 13 The Emergency Department, Copenhagen University Hospital, Hvidovre, Denmark; 14 Optimed, Clinical Research Centre, Copenhagen University Hospital, Hvidovre, Denmark; 15 PMR-C, Department of Physical and Occupational Therapy, Copenhagen, Denmark; 16 Section of Biostatistics, Department of Public Health, University of Copenhagen, Copenhagen, Denmark; 17 Department of Orthopaedic Surgery, Copenhagen University Hospital, Hvidovre, Denmark; 18 The Emergency Department, Copenhagen University Hospital, Hvidovre, Denmark; 19 Department of Physical and Occupational Therapy, Copenhagen University Hospital, Hvidovre, Denmark; 20 Department of Emergency Medicine, Odense University Hospital, Odense, Denmark; 21 Clinical Pharmacology and Pharmacy, Department of Public Health, University of Southern Denmark, Odense, Denmark; 22 Department of Emergency Medicine, Skåne University Hospital, Lund, Sweden; 23 Department of Emergency Medicine, Helsingborg Hospital, Helsingborg, Sweden; 24 Simulation Center (SimC), Odense University Hospital, Odense C, Denmark; 25 Radiology Department, Odense University Hospital, Odense C, Denmark; 26 Institute for Clinical Research, University of Southern Denmark, Odense C, Denmark; 27 Department of Emergency Medicine, Odense University Hospital, Odense C, Denmark; 28 Department of Respiratory Medicine, Odense University Hospital, Odense C, Denmark; 29 Copenhagen Academy for Medical Simulation and Simulation (CAMES), University of Copenhagen and the Capital Region of Denmark, Copenhagen, Denmark; 30 University of Southern Denmark, Odense, Denmark; 31 Department of Emergency Medicine, Hospital of South West Jutland, Esbjerg, Denmark; 32 University of Southern Denmark, Institute of Regional Health Research, Esbjerg, Denmark; 33 Department of Cardiology, Herlev-Gentofte Hospital, Copenhagen, Denmark; 34 Department of Emergency Medicine, Herlev-Gentofte Hospital, Copenhagen, Denmark; 35 Department of Anaesthesia, Center of Head and Orthopaedics, Rigshospitalet, University of Copenhagen, Copenhagen, Denmark; 36 Department of Emergency Medicine, Bispebjerg Hospital, Copenhagen, Denmark; 37 Department of Emergency Medicine, Odense University Hospital, Odense, Denmark; 38 Department of Respiratory Medicine, Odense University Hospital, Odense, Denmark; 39 Department of Clinical Biochemistry and Pharmacology, Odense University Hospital, Odense, Denmark; 40 Department of Emergency Medicine, Hospital of South West Jutland, Esbjerg, Denmark; 41 Emergency Department, Copenhagen University Hospital Bispebjerg and Frederiksberg, Copenhagen, Denmark; 42 The Emergency Department, Hospital of Southern Jutland, Aabenraa, Denmark; 43 Danish Institute of Regional Health Research, University of Southern Denmark, Odense C, Denmark; 44 The Emergency Department, Hospital of Southern Jutland, Aabenraa, Denmark; 45 Danish Institute of Regional Health Research, University of Southern Denmark, Odense C, Denmark; 46 Interdisciplinary Centre for Organizational Architecture, Business and Social Sciences Aarhus University, Aarhus C, Denmark; 47 DESIGN-EM - Research Network for Organization Design and Emergency Medicine, Aarhus, Denmark; 48 Department of Business Development and Technology, Aarhus University, Herning, Denmark; 49 Interdisciplinary Centre for Organizational Architecture, Business and Social Sciences, Aarhus University, Aarhus C, Denmark; 50 DESIGN-EM - Research Network for Organization Design and Emergency Medicine, Aarhus, Denmark; 51 Department of Business Development and Technology, Aarhus University, Herning, Denmark; 52 Optimed, Clinical Research Centre, Copenhagen University Hospital, Hvidovre, Denmark; 53 Acute Medical Unit, Copenhagen University Hospital, Hvidovre, Hvidovre, Denmark; 54 Paramedics, Greater Copenhagen Fire Department, Copenhagen, Denmark; 55 Department of Research and Development, Falck, Copenhagen, Denmark; 56 Department of Emergency Medicine, Hospital of South West Jutland, Esbjerg, Denmark; 57 Institute of Regional Health Research, University of Southern Denmark /Centre South West Jutland, Esbjerg, Denmark; 58 CT Innovation Unit, Department Of Radiology, Herlev Hospital, Herlev, Denmark; 59 Department Of Cardiology, Herlev Hospital, Herlev, Denmark; 60 Emergency Department, Herlev Hospital, Herlev, Denmark; 61 Department Of Gastrointestinal Surgery, Herlev Hospital, Herlev, Denmark; 62 University of Southern Denmark, Odense, Denmark; 63 University of Copenhagen, Copenhagen, Denmark; 64 Department of Emergency Medicine, Hospital of South West Jutland, Esbjerg, Denmark; 65 University of Aalborg, Aalborg, Denmark

## A1 QuickSOFA is an independent predictor of 30-day mortality among patients admitted to an emergency department with suspected or documented infection

### Osama Bin Abdullah^1^, Johannes Grand^1^, Astha Sijapati^1^, Petrine Nimskov^1^, Finn Erland Nielsen^1,2^

#### ^1^Department of Emergency Medicine, Slagelse Hospital, Slagelse, Denmark; ^2^Department of Clinical Research and Institute of Regional Health Services Research, University of Southern Denmark, Odense M, Denmark

##### **Correspondence:** Finn Erland Nielsen (fien@regionsjaelland.dk)

**Background**

Definitions and clinical criteria for sepsis have been revised in 2016. A simple bedside score (‘qSOFA’, for quick Sequential [Sepsis-Related] Organ Failure Assessment) has been proposed, which incorporates hypotension (systolic blood pressure ≤100 mmHg), altered mental status and respiratory rate ≥ 22/min: the presence of at least two of these criteria has been associated with poor outcomes typical of sepsis. The aim of this study was to evaluate qSOFA as a predictor of 30-day mortality in a model with other predictors of death among patients admitted to a single-centre emergency department (ED).

**Methods**

A historical cohort study among prospectively registered patients with suspected or documented infection. The admission period was from 1^st^ of November 2013 to 31^th^ of October 2014. Baseline clinical data and data for survival were obtained from a standard sepsis admission form, the patient records and The Danish Civil Registration System. Logistic regression analysis was used to adjust for potential confounders and to determine whether the predictive factors for death in the crude analyses were independently associated with 30-day mortality.

**Results**

A total of 434 patients were included in the study. Fifty-seven (13.1%; 95% confidence interval [CI] 9.9-16.3%) patients died during the first 30 days. Among several potential confounders tested in the model we found that age (odds ratio [OR] 1.29; 95% CI 1.03-1.61), Charlson Comorbidity Score ≥ 3 (OR 3.83; 95% CI 1.41-10.37), qSOFA score ≥2 (OR 4.78; 95% CI 2.09-10.91) and lactate values (lactate values < 2.0 as reference) within the interval 2.00-3.99 (OR 2.21; 95% CI 1.06-4.62) and lactate values ≥ 4.0 (OR 3.97; 95% CI 1.44-2,92) were associated with 30-day mortality.

**Conclusion**

qSOFA can be helpful to identify infectious patients in an ED with increased risk of 30-day mortality.

## A2 Patients' sense of safety in an emergency department – a qualitative study

### Jens Christian Schmidt^1^, Noel Pérez^2^, Tanja Kirkegaard^3^

#### ^1^Department of Anaesthesiology and Intensive Care Medicine, Hospitalsenheden Vest, Herning, Denmark; ^2^Emergency Department, Regionshospitalet Herning, Herning, Denmark; ^3^University of Aarhus, Arbejdsmedicinsk Klinik, Regionshospitalet Herning, Herning, Denmark

##### **Correspondence:** Noel Pérez (noelperez611@gmail.com)

**Background**

Feeling safe during hospitalization is essential. Several studies have indicated that feeling safe can counteract unnecessary hospital visits, reduce the length of hospital stay as well as avoid readmission and thereby reducing costs while improving care. Furthermore, it has been shown to improve compliance. This study aims to identify the most important factors affecting patients’ subjective experience regarding sense of safety when admitted to an emergency department (ED).

**Methods**

A qualitative case study design based on semi-structured individual interviews with adult patients (>18 years old) (n = 18) randomly selected and interviewed immediately after discharge from the ED. Interviews were digitally recorded and subsequently transcribed verbatim. Data collection and analysis were a combination of deductive and inductive strategies where the theoretical frame of reference and previous research guided the base structure of interviews. An inductive analysis of the data were conducted following the basic principles of grounded theory and categorized into concepts using open coding and subsequently organized by themes, which laid the ground for the development of stable categories.

**Results**

Five main categories were identified: (a) communication; (b) information; (c) safety-ensuring actions; (d) relatives; (e) busyness. Across categories, patients emphasized the importance of being taken seriously by the doctors and stressed clear language (ordinary language without foreign or latin words) as being important for their sense of safety as it enabled them to understand what the doctor was explaining and thereby enabled them to have a meaningful conversation and ask relevant questions. Patients also accentuated the importance of the doctors being ‘down-to-earth’, being calm and not showing signs of stress.

**Conclusions**

Research focusing on patients' perception of safety in EDs is limited, but seems to correlate well with findings in studies regarding inpatient hospitalization in general. Traditionally, literature has focused mainly on nurses’ role in making patients feel safe. This study, however, finds that the interaction with the doctors is equally important. The role of the doctor in relation to patients' sense of security when admitted to an ED is crucial and further studies are needed.

## A3 Acutely admitted medical patients have increasing one-year mortality with increasing age, a follow up study

### Marianne Fløjstrup^1^, Mikkel Brabrand^2,3,4^

#### ^1^Department of anaesthesiology, Vejle Sygehus, Vejle, Denmark; ^2^Medical Admission Unit 272, Hospital of South West Jutland, Esbjerg, Denmark; ^3^Emergency Department, Odense University Hospital, Odense, Denmark; ^4^Institute of Regional Health, University of Southern Denmark, Esbjerg, Denmark

##### **Correspondence:** Marianne Fløjstrup (flojstrup1@hotmail.com)

**Background**

The majority of patients admitted to a medical admission unit are old. Previous international studies have shown increased one-year mortality with increasing age. The aim of this study is to clarify if one-year mortality of Danish medical patients increases with age.

**Methods**

We followed a cohort of consecutive, acutely admitted adult (15+ years) medical patients for one year after admission. The patients were admitted from June 2012 to November 2012 to the medical admission unit at The Hospital of South West Jutland, Esbjerg. Only patients who were not Danish residents were excluded. We extracted survival status from the Danish Civil Register and calculated mortality at one year stratified by age in the groups 15-49, 50-64, 65-79 and 80+ years.

We calculated both crude hazard ratios using Cox Proportional Hazard Regressions analysis and adjusted for potential confounders: gender, Charlson comorbidity index (CCI) and vital signs at time of admission. Data will be presented as median (range) or proportion (95% confidence interval) unless otherwise stated.

**Results**

5784 patients were admitted, median age 67 (15-101) years and 2917 (47%) were female.

The crude one-year all-cause mortality was 17.7 (16.8-18.7) %. The one-year mortality of patients 15-49 years was 1.6 (1.1-2.4) %, 50-64 years 8.7 (7.2-10.5) %, 65-79 years 22.0 (20.2-24.0) % and 80+ years 38.0 (35.4-40.6) %.

Crude Hazard ratio for patients 50-64 years old was 5.7 (3.6-9.0), for 65-79 year olds 15.5 (10.2-23.5), and 80+ years 30.0 (19.7-45.6) compared to patients 15-49 years old.

Adjusting for gender, CCI and vital signs, hazard ratios for patients 50-64 years old was 4.6 (2.2-9.6), for 65-79 year olds, it was 10.2 (5.2-20.2) and 80+ years 20.6 (10.4-40.5) compared to patients 15-49 years old.

**Conclusion**

One-year mortality for acutely admitted medical patients increased almost linear with increasing age.

## A4 Use of intravenous antibiotics decrease by daily focus of indication

### Mathias Galthen-Sørensen^1^, Rasa Ramoskiene^1^, Arman Arshad^1^, Annmarie Lassen^2^, Lars Stubbe Teglbjærg^1^

#### ^1^Department of Internal Medicine, Odense University Hospital, Svendborg, Denmark; ^2^Department of Emergency, Odense University Hospital, Odense, Denmark

##### **Correspondence:** Mathias Galthen-Sørensen (mathias.galthen-sorensen@rsyd.dk)

**Background**

Use of antibiotics has over time resulted in decrease in morbidity and mortality. An increasing use has however resulted in resistance and super-infections. There is consensus that use of antibiotics needs to be reduced to prevent a post-antibiotic era.

**Methods**

The project was conducted in five departments (i.e. emergency department, cardiology, gastroenterology, pulmonology, and rheumatology) at Odense University Hospital, Svendborg, in the period 1^st^ of April 2016 to 30^th^ of April 2016. During the project period a single doctor reviewed ordinations and made suggestions to change in treatment if prescription was not in line with local guidelines. Use of antibiotics was compared to the mean monthly use the year before. At first and last day of the project, stocks of intravenous antibiotics were stated for all departments. Retrospectively, purchases of intravenous antibiotics were drawn for the project period as well as for the time period one year before. Doctors were informed about the project as a monthly “point of focus” prior to project start and reminded on procedures when prescribing antibiotics. Data was analysed with STATA and amount of units used as well as cost was compared with the month mean for the year prior of the project period.

**Results**

Among all antibiotics, an overall decrease in used doses (UD) was observed (1489 vs. 2479 UD, 40%). For the groups of 2nd, 4th generation penicillins, carbapenems and cephalosporin’s a decrease was seen (71%, 46%, 88% and 25%) while for vancomycin no units were used compared to the monthly mean usage of 30 units the year ahead. For the narrow spectrum penicillin a decrease in usage was observed too (penicillin G 18% and dicloxacillin 84%) and metronidazol usage was reduced with 21%. In contrast an increase in aminoglycosides and flourquinolones was observed (46% and 49%). Antibiotic costs were reduced with 41%.

**Conclusions**

By focusing on the guidelines of prescribing antibiotics and a daily review of all admitted patients it is possible to reduce the amount of most types of antibiotics used, especially those of broad spectrum.

## A5 A screening tool: Info-65 implemented in the emergency department targeting readmission of elderly acute admitted medical patients in a Danish hospital

### O Andersen^1^, L Mørch Jørgensen ^2^, D M Sivertsen^1^, J W Kirk^1^, J Petersen^1^

#### ^1^Optimed, Clinical Research Centre, Copenhagen University Hospital, Hvidovre, Denmark; ^2^The Emergency Department, Copenhagen University Hospital, Hvidovre, Denmark

##### **Correspondence:** O Andersen (ove.andersen.privat@gmail.com)

**Background**

Older patients, age +65 years are at increased risk of being admitted to an emergency department (ED). Moreover, 30% of elderly acutely admitted patients are readmitted within 3 months. This is a challenge socioeconomically, and physically and psychologically for the patient. The aim of this study was: to implement and test a screening tool targeting readmission within 90 days of elderly in collaboration between the hospital and municipalities.

**Methods**

A pilot implementation study with inclusion criteria: 65+ years old, admitted to the medical section of the ED at a 600 bed University Hospital and living in Copenhagen, Brøndby or Hvidovre municipalities. Exclusion were; not able to cooperate cognitively or physically, a cancer diagnosis, not Danish speaking or discharged prior to screening. Patients were screened within 24 hours after admission by the Geriatric Team in the ED with a screening tool previously developed, consisting of three elements: routine blood tests indicative for the presence of low-grade inflammation (suPAR) and infection (CRP); three questions examining: (1) help at home, (2) times out of the home and (3) hospitalization within the last 6 months; and a 4-m walking test.

**Results**

From 1^st^ of September 2013 to 1^st^ of July 2014, 3666 patients fulfilled the inclusion criteria; of these 1506 (41%) were seen by the Geriatric team and 38% of these were subsequently excluded. The Geriatric Team fulfilled the whole screening on 811 patients. Of the screened patients, the screening tool showed that 537 (66%) were at higher risk of being readmitted within 90 days. This high-risk group had a 50% higher hazard of readmission within 3 month than the “low-risk” group, (Hazard Ratio = 1.5 (CI: 1.1-2.0), p = 0,005). The cumulated risk of readmission was 42% in the high-risk group and 30% in the low-risk group.

**Conclusion**

The screening tool was successfully implemented in the ED and was able to predict 90 days re-admittance rate in acutely admitted +65 years old patients.

## A6 Exploring how inflammation underlies adverse health outcomes in acutely admitted older medical patients; associations between different inflammatory patterns, and physical- and organ function

### H H Klausen^1^, A C Bodilsen^1,2,6^, J Petersen^1,3^, T Bandholm^1,2,4,6^, T Haupt^1^, DM Sivertsen^1^, O Andersen^1,5^

#### ^1^Optimed, Clinical Research Centre, Copenhagen University Hospital, Hvidovre, Denmark; ^2^PMR-C, Department of Physical and Occupational Therapy, Copenhagen, Denmark; ^3^Section of Biostatistics, Department of Public Health, University of Copenhagen, Copenhagen, Denmark; ^4^Department of Orthopaedic Surgery, Copenhagen University Hospital, Hvidovre, Denmark; ^5^The Emergency Department, Copenhagen University Hospital, Hvidovre, Denmark; ^6^Department of Physical and Occupational Therapy, Copenhagen University Hospital, Hvidovre, Denmark

##### **Correspondence:** O Andersen (ove.andersen.privat@gmail.com)

**Background**

In the general population, inflammation is associated with age-related physical performance and organ function. This is unknown in acutely admitted older medical patients.

Aim: Firstly, to investigate if systemic inflammation in acutely admitted older medical patients is associated with physical performance, and organ dysfunction. Secondly, to investigate if the association between organ dysfunction and physical performance is mediated by systemic inflammation.

**Methods**

A cross sectional cohort study of medical patients (65+) admitted to an Emergency Department. Physical performance was assessed by four meter gait speed, handgrip strength and the de Morton Mobility Index (DEMMI), and organ dysfunction by the number of standard laboratory tests outside the reference range (OutRef). Systemic inflammation was assessed by concentrations of IL-6, TNFα and suPAR. Associations were investigated by multiple regression analyses, adjusted for age, sex, cognitive impairment, and severity of acute illness, estimated by CRP and VitalPAC Modified Early Warning Score (ViEWS).

**Results**

The cohort included 369 patients with a median age of 77.9 years. In adjusted analyses, IL-6 was associated with handgrip strength (p = 0.007); TNFα with DEMMI (p < 0.001) and handgrip strength (p = 0.004), and suPAR with all physical performance measurements (p < 0.001). All three inflammation markers were associated with OutRef (p < 0.001). OutRef was associated with all physical performance measurements (p < 0.001) in analyses adjusted for age, sex, cognitive impairment and ViEWS.

**Conclusion**

Systemic inflammation seems to be mediating both organ dysfunction and low physical performance in acutely admitted older medical patients and thus could be a clinical feasible modality for systematic assessment of vulnerability in this population.

## A7 QTc prolongation and prognosis among patients with suspected poisoning in the emergency department – a propensity score matched cohort study

### Camilla Schade Hansen^1^, Anton Pottegård^2^, Ulf Ekelund^3^, Jakob Lundager Forberg^4^, Helene Kildegaard Jensen^1^, Annmarie Touborg Lassen^1^

#### ^1^Department of Emergency Medicine, Odense University Hospital, Odense, Denmark; ^2^Clinical Pharmacology and Pharmacy, Department of Public Health, University of Southern Denmark, Odense, Denmark; ^3^Department of Emergency Medicine, Skåne University Hospital, Lund, Sweden; ^4^Department of Emergency Medicine, Helsingborg Hospital, Helsingborg, Sweden

##### **Correspondence:** Camilla Schade Hansen (Camilla.Schade.Hansen@rsyd.dk)

**Background**

Poisoning is a frequent cause of admission to the emergency department (ED), which may involve drugs known to prolong the QT interval. The aims of this study were to describe the prevalence of QTc prolongation among ED patients with suspected poisoning and to calculate within this population the absolute and relative risk of 30-day mortality or cardiac arrest associated with a prolonged QTc interval.

**Methods**

We performed a register-based cohort study, including all adult first time admissions with suspected poisoning to the ED of Skåne University Hospital, Lund and Helsingborg Hospital (January 2010 to December 2014), or Odense University Hospital and the Hospital of South West Jutland (March 2013 to April 2014). We used propensity score matching to calculate hazard ratios for all-cause mortality or cardiac arrest within 30 days after admission comparing patients with a prolonged QTc interval (≥450 ms men, ≥460 ms women) with patients with a QTc interval of <440 ms.

**Results**

Among all first time admissions with suspected poisoning that had an ECG recorded (n = 3869), QTc prolongation occurred in 6.5% (95% CI, 5.8-7.4) with the highest prevalence among those poisoned by drugs categorized as “others” (6.4%; 95% CI, 3.9-9.7). The overall mortality or cardiac arrest after a 30-day follow-up period was 0.8% (95% CI, 0.5-1.2), with an absolute risk of event in patients with QTc prolongation of 3.2% (95% CI, 1.4-6.1). A prolonged QTc interval on admission was associated with a HR of 2.9 (95% CI, 0.9-9.1).

**Conclusion**

In the ED, a prolonged QTc interval in patients arriving with suspected poisoning is associated to an almost 3-fold increase in 30-day all-cause mortality or cardiac arrest. However, the absolute risk is low.

## A8 Learning curves for focused lung ultrasound in the hands of emergency physicians

### Janni Lynggård Bo Madsen^1^, Ole Graumann^2,3^, Stefan Posth^3,4^, Pia Iben Pietersen^5^, Lars Konge ^6^, Christian B. Laursen^3,5^

#### ^1^Simulation Center (SimC), Odense University Hospital, Odense C, Denmark; ^2^Radiology Department, Odense University Hospital, Odense C, Denmark; ^3^Institute for Clinical Research, University of Southern Denmark, Odense C, Denmark; ^4^Department of Emergency Medicine, Odense University Hospital, Odense C, Denmark; ^5^Department of Respiratory Medicine, Odense University Hospital, Odense C, Denmark; ^6^Copenhagen Academy for Medical Simulation and Simulation (CAMES), University of Copenhagen and the Capital Region of Denmark, Copenhagen, Denmark

##### **Correspondence:** Janni Lynggård Bo Madsen (jamad12@student.sdu.dk)

**Background**

Focused ultrasound is often described as having a shallow learning curve and many guidelines recommend a certain number of examinations needed to obtain competency. The aim of this study was to determine learning curves for focused lung ultrasound (FLUS) performed by physicians after completing a theoretical and practical course.

**Methods**

The ultrasound course was comprised of a both theoretical and interactive 4-6 hours e-learning course and a two-day hands-on course. Physicians were included if they in the period November 2013 to November 2015, had completed a course in focused emergency medicine ultrasound and subsequently uploaded 25 FLUS examinations for assessment by an expert in diagnostic LUS. The result of an uploaded FLUS examination was defined as being successful if the course participant correctly had identified the presence or absence of pneumothorax, pleural effusion, and interstitial syndrome. The expert assessment of the FLUS examination was used as the reference test. The learning curves were calculated using cumulative sum (CUSUM) analysis. Acceptable and unacceptable failure rates were designated as being 5% and 10%, respectively.

**Results**

A total of 12 physicians having uploaded 25 LUS examinations were identified. The CUSUM scores for each of the 12 physicians were calculated. Nine of the physicians had CUSUM learning curves with a relatively downward or stable projection as a sign of developing competence. Three physicians, however, had CUSUM curves with an upward projection indicating unacceptable competence in performing the procedure.

**Conclusion**

The learning curves for FLUS performed by physicians differ between trainees and are not necessarily shallow. Our findings imply that competency in the examination following a theoretical and practical course is not adequate. Based on the results, we cannot recommend using a predefined number of examinations in order to obtain competency in FLUS. Assessment tools with solid evidence of validity are needed to assess competency in FLUS.

CUSUM score analysis could serve as a clinical tool to identify physicians needing additional training in the procedure.

## A9 Quick SOFA, rapid emergency medicine score and national early warning score can all identify acutely admitted medical patients at increased risk of 7-day mortality

### Janni Lynggård Bo Madsen^1^, Søren Nygaard Hansen^1^, Kristian Møller Jensen^1^, Mikkel Brabrand^2,3^

#### ^1^University of Southern Denmark, Odense, Denmark; ^2^Department of Emergency Medicine, Hospital of South West Jutland, Esbjerg, Denmark; ^3^University of Southern Denmark, Institute of Regional Health Research, Esbjerg, Denmark

##### **Correspondence:** jamad12@student.sdu.dk

**Background**

It is inherently difficult to assess how sick a patient is. Most staff develops the ability to differentiate between sick and less sick patients over time, but experience takes time. Thus we require other ways to identify high-risk patients in hospitals. Several scoring systems have been developed. In this abstract we present an external validation of the quick SOFA (qSOFA), Rapid Emergency Medicine Score (REMS) and the National Early Warning Score (NEWS).

**Methods**

We included all first-time acute admissions of adult (≥15 years) patients to the acute medical unit at The Hospital of South West Jutland from 1^st^ of June 2012 to 1^st^ of November 2012. We used the first vital signs measured upon arrival to the unit and calculated the scores according to the original papers. The endpoint was 7-day all-cause mortality, which was extracted from the Danish Civil Registration System. Data will be presented as median (range) or proportion (95% confidence interval). The ability to identify patients at increased risk (discriminatory power) will be presented as area under the receiver operating characteristics curve (AUROC). The discriminatory power of the scores was compared according to Hanley and McNeil.

**Results**

We included 5,784 patients, median age was 67 (15-101) years and 2,917 (50.4%) were female. 111 (1.9%) patients met the endpoint. We were able to calculate qSOFA for 2,872 (49.7%) patients. AUROC for 7-day mortality for qSOFA was 0.733 (0.644-0.823). We could calculate REMS for 2,706 (46.8%) patients. AUROC for 7-day mortality for REMS was 0.787 (0.709-0.845). We could calculate NEWS for 2,708 (46.8%) patients. AUROC for 7-day mortality for NEWS was 0.808 (0.714-0.901). We were able to calculate both qSOFA, REMS and NEWS for 2,706 patients and found no significant difference in discriminatory power, p = 0.55.

**Conclusion**

In this single centre retrospective study, using 7-day all-cause mortality as endpoint, we found that both qSOFA, REMS and NEWS were able to identify patients at increased risk, but only NEWS with adequate reliability.

## A10 Does clinical assessment by emergency department nurses change the triage level of patients

### Rasmus Bo Hasselbalch^1^, Mia Pries-Heje^1^, Lisbet Ravn^2^, Morten Lind^2^, Lars S. Rasmussen^3^, Birgitte Nybo Jensen^4^

#### ^1^Department of Cardiology, Herlev-Gentofte Hospital, Copenhagen, Denmark; ^2^Department of Emergency Medicine, Herlev-Gentofte Hospital, Copenhagen, Denmark; ^3^Department of Anaesthesia, Center of Head and Orthopaedics, Rigshospitalet, University of Copenhagen, Copenhagen, Denmark; ^4^Department of Emergency Medicine, Bispebjerg Hospital, Copenhagen, Denmark

##### **Correspondence:** Rasmus Bo Hasselbalch (r.hasselbalch@gmail.com)

**Background**

Triage in an emergency department (ED) is used to identify patients that should be treated first. The Copenhagen Triage Algorithms (CTA) is a new quicker triage model based on vital signs and a clinical assessment by the ED nurse. In this model, the ED nurse can reclassify patients as more or less urgent based on a clinical assessment. Our aim was to see how this option affected final triage level.

**Methods**

The CTA study is a prospective two-centre, cluster-randomized, cross-over, non-inferiority trial comparing CTA to the standard triage model (DEPT). All ED admissions of patients ≥16 years at two large hospitals were included. Centres are randomly assigned to perform either CTA or DEPT triage until half of the sample size is included and subsequently cross-over. Both models stratify patients in 4 acuity levels, while patients with minor injuries are excluded.

**Results**

A total of 54,249 patient admissions were recorded during the study period. Of these 48,762 (90%) patients were included in the analysis and 24,759 (51%) were triaged with CTA. The clinical assessment changed the triage level in 10,334 (43%) patients. Most of these, 9,118 (88%), were reclassified as more urgent. Significant predictors of this were lower age, lower respiratory rate, and lower heart rate, higher arterial oxygen saturation and higher systolic blood pressure (all P < 0.001). A total of 1,216 (12%) were reclassified as less urgent. Significant predictors of this were higher age, higher respiratory rate, higher heart rate, lower arterial oxygen saturation and lower systolic blood pressure (all P < 0,001).

**Conclusion**

The option to change the triage level was used frequently. Most of reclassifications were to a more urgent level of triage in patients where vital signs seemed to be less concerning.

## A11 Validity of the qSOFA score to identify severely ill patients among medical patients with suspected infection in an Emergency Department - A follow-up study at Odense University Hospital 2012-2015

### Ulrik Havshøj^1^, Daniel Pilsgaard Henriksen^2,3^, Mikkel Brabrand^1,4^, Annmarie Touborg Lassen^1^

#### ^1^Department of Emergency Medicine, Odense University Hospital, Odense, Denmark; ^2^Department of Respiratory Medicine, Odense University Hospital, Odense, Denmark; ^3^Department of Clinical Biochemistry and Pharmacology, Odense University Hospital, Odense, Denmark; ^4^Department of Emergency Medicine, Hospital of South West Jutland, Esbjerg, Denmark

##### **Correspondence:** Ulrik Havshøj (emailulrik.havshoej@rsyd.dk)

**Background**

Sepsis is a common and severe condition. As prompt treatment improves prognosis, sepsis is considered a time dependent disease. Therefore, it is important to have a reliable scoring system to quickly identify the severely ill septic patients on arrival. The recently introduced qSOFA score (≥2 of: Systolic blood-pressure ≤ 100 mm Hg, Glasgow coma score <15 and respiratory rate ≥22/min) is aimed at doing so. In this study, we present an external validation of qSOFA in an ED setting among medical patients.

**Method**

We conducted a follow up of all adult (≥18 years) patients admitted to the ED at Odense University Hospital from 1^st^ of April 2012 to 31^th^ of March 2015. We included the first ED contact within the inclusion period, where blood cultures were drawn. The vital signs were measured on the patients’ arrival. Our combined endpoint was death, or spending ≥ three days in the ICU, during admission, in accordance with the original criteria. All data were analyzed using STATA version 14.0.

**Results**

Among the 212,703 ED contacts in the period, blood cultures were drawn on 11,835 (9.0%) medical contacts. We included 8,750 (4.1%) individuals in our study, but due to missing data 7,970 (91.0%) were eligible for analysis. Of these, 5,134 (64.4%) had a qSOFA score of 0, 2,365 (29.7%) of 1,423 (5.3%) of 2 and 48 (0.6%) of 3.690 (8.6%) met our endpoint (137 (19.9%) with a positive and 553 (80.1%) with a negative qSOFA score). Sensitivity was 19.9% (16.9-23.0%) and specificity 95.9% (94.9-95.9%). The positive predictive value was 29.1% (25.0-33.4%) and negative predictive value 92.6% (92.0-93.2%). Area under Receiver Operating Curve was 0.68.

**Conclusion**

One third of all patients with suspected infection admitted to an ED with a positive qSOFA score either died during their admission or were transferred to the ICU within three days after admission. Despite of a high specificity, qSOFA failed to identify four out of five of the patients with the worst prognosis.

## A12 Comparative study of the temporal artery thermometer vs the rectal thermometer

### Hanne H Nygaard, Christian Maschmann

#### Emergency Department, Copenhagen University Hospital Bispebjerg and Frederiksberg, Copenhagen, Denmark

##### **Correspondence:** Hanne H Nygaard (hanne.helleskov.nygaard@regionh.dk)

**Background**

The body temperature is an important parameter for the initial diagnostic, clinical decisions and care. In the Emergency Department (ED) and when hospitalized, frequent temperature measurements are important to identify the development of critical illness. Therefore, prompt and accurate equipment for measuring body temperature is necessary. Various non-invasive and easily used digital thermometers are available including the Temporal Artery Thermometer (TAT). However, there is lack of evidence for using the TAT-measurement as an accurate non-invasive method for measuring body temperature.

**Methods**

We designed a prospective comparative study of body temperature measurements using the TAT (Exergen TAT-5000 from Exergen Corporation, Watertown, Massachusetts, USA) and a conventional digital rectal thermometer (Omron MC-341-E, OMRON healthcare Europe B.V., Hoofddroop, Holland), respectively. To eliminate interpersonal variations all measurements were conducted by one nurse specialist responsible for the data collection. Data were collected at the ED, Bispebjerg Hospital, Denmark. During Marts-June 2013, N = 381 patients ≥18 years were enrolled. Patients <18 years or patients with constipation or anal disorders were excluded. Bland Altman difference plot was used to compare and analyse the two measurements. The maximum allowed difference between the two measurements was set to +/- 0.3 °C. In addition, the sensitivity and specificity for the TAT was calculated.

**Results**

The maximum and minimum differences measured were +1.7 and -1.7 °C and the mean of the differences were 0.17 (SD = 0.47). 53% (N = 202) of the paired measurement deviated less than +/-0.3 °C. The sensitivity of the TAT to detect fever was 66.7% (CI: 49.0–81.4), the specificity was 95.9% (CI: 93.3–97.8). The positive and negative predictive value was 63.2% (CI: 46.0–78.2) and 96.5% (CL: 94.0–98.2), respectively.

**Conclusions**

The study showed inacceptable wide temperature deviations between measurements performed with the TAT compared with the rectal measurements being performed with a conventional rectal thermometer. Furthermore, the capability of the TAT detecting fever was unacceptably low.

Based on these study results the use of the TAT cannot be recommended as an accurate screening tool for temperature measurements in the ED.

## A13 Are prehospital providers measurements of normal vital sign predictive of emergency nurses’ measurements?

### Helene Skjøt-Arkil^1,2^, Christian Backer Mogensen^1,2^

#### ^1^The Emergency Department, Hospital of Southern Jutland, Aabenraa, Denmark; ^2^Danish Institute of Regional Health Research, University of Southern Denmark, Odense C, Denmark

##### **Correspondence:** Helene Skjøt-Arkil (Helene.Skjoet-Arkil@rsyd.dk)

**Background**

Vital signs are an important part of the hospital triage algorithms, which prioritizes patients according to urgency of treatment. Most vital signs are assessed by the nursing staff on arrival to the emergency department (ED). In the previous years, studies have focused on the possibilities of a uniform triage and investigated the agreement between pre-hospital providers and ED personnel. However little is known about the agreement of the recordings of normal vital sign ranges between pre-hospital providers and ED nurses. The aim of the study was to investigate if the normal vital sign ranges measured by the pre-hospital providers are consistent with the recorded values of the ED nurses.

**Methods**

The study was prospective and observational. Patients transported to the ED by ambulances were included. Vital signs were measured by pre-hospital providers on arrival to the hospital, and by ED nurses upon arrival to the ED. Normal vital signs ranges were: Respiratory rate (RR) < 20 breaths/min, Glasgow Coma Score (GSC) = 15, saturation of peripheral oxygen (SpO_2_) > 94, heart rate (HR) ≥60 and ≤100 beats/min, systolic blood pressure (SBP) >90 and <160 mmHg, temperature (Tp) < 38 °C. An agreement analysis was conducted and Bland-Altman plots were performed.

**Results**

292 patients were included in the study. Pair wise analyses showed small mean differences RR: -0.4 (CI -0.8-0.0) breaths/min, HR: -1.4 (CI -2.4-(-0.4)) beats/min, SpO_2_: 0.3 (CI 0.0-0.5) %, SBP: 1.4 (CI -0.7-3.4) mmHg and Tp: 0.0 (CI -0.1-0.1) °C. Bland-Altman comparisons and plots demonstrated the following limits of agreement RR: -5 to 4 breaths/min, HR: -15 to 12 beats/min, SpO_2_: -4 to 4%, SBP: -30 to 32 mmHg and Tp: -0.9 to 0.9 °C.

**Conclusions**

The study showed no clinical important differences in overall mean of the vital signs recorded by pre-hospital providers and ED nurses for normal ranges. The limits of agreement were however larger. Normal vital signs measured by the pre-hospital providers are only to a moderate degree predictive of the emergency nurses’ measurements.

## A14 Evaluation of the Danish Regions Pediatric Triage model

### Lotte Høeg Hansen^1^, Lena Wittenhoff^1^, Christian Backer Mogensen^1,2^, Helene Skjøt-Arkil^1^

#### ^1^The Emergency Department, Hospital of Southern Jutland, Aabenraa, Denmark; ^2^Danish Institute of Regional Health Research, University of Southern Denmark, Odense C, Denmark

##### **Correspondence:** Helene Skjøt-Arkil (Helene.skjoet-arkil@rsyd.dk)

**Background**

The critically ill children are not always recognized when they enter the pediatric department, even when assessed by experienced pediatric nurses. Pediatric triage is used worldwide to differentiate the critically ill patients who need immediate attention from patients who can safely wait.

The Danish Regions Pediatric Triage model (DRPT) was introduced in 2012 and has now been implemented in all Danish acute pediatric departments. The DRPT has not been validated so far. We thus aimed to evaluate the validity of DRPT as a screening tool to detect both the most serious acute conditions and the non-serious conditions in daily clinical use in a pediatric department.

**Methods**

A prospective cohort study with follow-up of children with acute referral to pediatric ward from October to December 2015. Two proxy-methods was used to determine the true urgency: a reference standard and real outcomes in terms of admission to hospital, admission to intensive care unit, return to home without any treatment and utilized resources. We made four analysis: The DRPT ability to detect (I) children with the most urgent needs (II) children who had no urgent needs according to the reference model (III) children who actually had a life threatening condition (IV) children who were returned home without any treatment. For all these analysis we calculated the sensitivity, specificity, positive predictive value, negative predictive value, accuracy and likelihood for positive and negative test, all with a 95% confidence interval.

**Results**

We included 550 patients. The DRPT agreed with the reference standard in 166 patients (30%). DRPT under-triaged in 296 of the children (54%) and over-triaged in 88 patients (16%). The sensitivity of the triage to detect very urgent patients was 36%. Among the non-urgent children the triage had a sensitivity of 50% and a negative predictive value of 90%.

**Conclusion**

The DRPT is a weak triage system to identify the few, sickest children, who need immediate care as well as the many children who can be sent home with no further intervention.

## A15 The collaboration between the emergency department and the other departments at the hospital as a possible explanation of the weekend effect in an emergency department

### Iben Duvald^1,2,3^ (idp@icoa.au.dk)

#### ^1^Interdisciplinary Centre for Organizational Architecture, Business and Social Sciences, Aarhus University, Aarhus C, Denmark; ^2^DESIGN-EM - Research Network for Organization Design and Emergency Medicine, Aarhus, Denmark; ^3^Department of Business Development and Technology, Aarhus University, Herning, Denmark

**Background**

Studies have shown that acute patients admitted to hospitals during weekends experience worse outcomes than those admitted on a weekday (i.e. 30-days mortality, length of stay and number of adverse events). However, it is debated why these variations occur. This study explores whether the collaboration between the emergency department and the other departments at the hospital can explain the existence of this weekend effect in an emergency department.

**Methods**

The study is a prolonged six-month ethnographic study. Fieldwork consisted of app. 700 hours of participant observations in an emergency department, 21 in-depth interviews with nurses, physicians, secretaries and the management of the department and two focus groups with eight nurses. The empirical material was generated in autumn 2015 and spring 2016 in a medium-sized regional hospital located in Denmark. In order to provide sufficient care and treatment for in-coming patients, emergency departments are highly dependent on the other departments at the hospital. The results of this study identified different challenges in the collaboration between the emergency department and the other departments at the hospital. These challenges includes: (1) the transfer of patients to specialized departments; (2) the different ways the specialists from other departments are working in the emergency department and how this was structured; (3) the communication within the emergency department and between the departments; (4) accessing service departments at the hospital.

**Results and conclusion**

The findings showed that the collaboration between the emergency department and the other departments are organized in different ways, which results in different challenges especially coordination challenges. Furthermore, the emergency department could not always transfer the patients to the specialized departments, and the access to the service departments was limited in weekends. All these challenges can be possible explanations of the existence of the weekend effect in the emergency department.

## A16 Adverse events in an emergency department weekdays and weekends

### Iben Duvald^1,2,3^ (idp@icoa.au.dk)

#### ^1^Interdisciplinary Centre for Organizational Architecture, Business and Social Sciences, Aarhus University, Aarhus C, Denmark; ^2^DESIGN-EM - Research Network for Organization Design and Emergency Medicine, Aarhus, Denmark; ^3^Department of Business Development and Technology, Aarhus University, Herning, Denmark

**Background**

Admission to the hospital on weekends has been associated with a higher risk of adverse events. However, limited information is available concerning this “weekend effect”. This study investigated numbers and types of adverse events occurring in an emergency department weekdays and weekends, and how the employees in the emergency department relate to these adverse events.

**Methods**

This study followed a mixed-methods design consisting of 1) a prospective descriptive observational study and 2) qualitative interviews. The prospective descriptive observational study consists of two hundred twenty-nine adverse events, occurred in an emergency department at a Danish regional hospital, reported to the mandatory national reporting system during a two-year period (2014-2015). Moreover two focus groups with eight nurses, an in-depth interview with a physician and unstructured interviews with different employees have been conducted during the last year.

**Results**

The analysis showed that most adverse events happen on weekdays (0.13 per shift) when compared to weekends (0.07 per shift). But the results of this study also showed that 25% (57/229) of the reported adverse events occurred in weekends. Different types of adverse events happen weekdays and weekends. Most of the adverse events happen on weekdays are related to 1) samples, patient examination and test results and 2) medication. Adverse events related to 3) treatment and nurse care and 4) information handover, patient responsibility and documentation happen more often in weekends than on weekdays. Furthermore most adverse events happen between 2 pm and 4 pm. The focus group interviews suggest that employees perceive reported adverse events as a tool to optimize the work processes and quality of patient care provided in the emergency department. However, because of workload they did not report all adverse events which creates bias in the register data.

**Conclusion**

This study presented different tendencies in when and what kinds of adverse events are happening in an emergency department weekdays and weekends. Additionally, when only some adverse events are reported to the mandatory national reporting system this study also indicated problems in registration practices.

## A17 suPAR as a standard prognostic test in the acute medical unit at Hvidovre Hospital

### Line Jee Hartmann Rasmussen^1^, Steen Ladelund^1^, Thomas Huneck Haupt^1^, Gertrude Ellekilde^2^, Jesper Eugen-Olsen^1^, Ove Andersen^1^

#### ^1^Optimed, Clinical Research Centre, Copenhagen University Hospital, Hvidovre, Denmark; ^2^Acute Medical Unit, Copenhagen University Hospital, Hvidovre, Hvidovre, Denmark

##### **Correspondence:** Line Jee Hartmann Rasmussen (line.jee.hartmann.rasmussen@regionh.dk)

**Background**

The inflammatory biomarker soluble urokinase plasminogen activator receptor (suPAR) is a marker of disease, disease severity, and mortality. Since November 2013, suPAR has been a standard admission blood test in the acute medical unit (AMU) at Hvidovre Hospital. Here, we analyze the prognostic value of suPAR with regard to readmission and survival.

**Methods**

This registry-based cohort study includes 17,312 patients admitted to the AMU, Hvidovre Hospital, between November 18th 2013 and September 30th 2015. Patients were followed for 30 days and suPAR measurements were combined with data from national registries, including information on admissions, diagnoses, vital status etc. The Charlson Comorbidity score was calculated based on registered diagnoses as a measure of comorbidity burden. Kruskal-Wallis test and receiver operating characteristic (ROC) curve analysis was used for statistical analysis.

**Results**

Median suPAR for the 17,312 patients was 2.8 ng/ml (IQR: 1.9-4.3). Women had slightly higher suPAR compared with men (P < 0.0001) and suPAR increased with age (P < 0.0001). A high suPAR level was associated with longer in-hospital stays (P < 0.0001) and a higher Charlson Comorbidity score (P < 0.0001). Patients readmitted within 30-days follow-up had higher suPAR (3.5 ng/ml, IQR 2.3-5.3) compared with patients who were not readmitted and survived (2.6 ng/ml, IQR: 1.9-3.8). Similarly, patients who died within 30 days (n = 859, 6.2 ng/ml, IQR: 4.3-9.2) had significantly higher suPAR compared with patients who survived (2.7 ng/ml, IQR: 1.9-4.0, P < 0.0001). Combined ROC curve analysis for age, sex, and suPAR for 30-day mortality yielded an AUC of 0.88 (95% CI: 0.85-0.87) which was significantly better than age and sex alone (0.79, 95% CI: 0.78-0.80, P < 0.0001).

**Conclusions**

This large cohort study in the AMU verifies previous findings of suPAR as a strong risk marker in acute medical patients. In addition to disease severity and comorbidity burden, suPAR is associated with adverse outcomes, such as readmission and mortality and thus provides prognostic information.

## A18 Capnography and clinical decision making in the spontaneously breathing, non-intubated emergency patient - a systematic review

### Martin Betzer^1^, Rasmus Lyngby^2^

#### ^1^Paramedics, Greater Copenhagen Fire Department, Copenhagen, Denmark; ^2^Department of Research and Development, Falck, Copenhagen, Denmark

##### **Correspondence:** Martin Betzer (martin.betzer@gmail.com)

**Background**

The critical relationship of EtCO_2_ monitoring and clinical decision-making in intubated patient is well documented. The rationale and benefits from applying EtCO_2_ monitoring in the non-intubated cohort is, however, not well documented and guidelines and recommendations for its application are sparse. The aim of this systematic review was to investigate the possible benefits of capnography to clinical decision making in the spontaneously breathing, non-intubated emergency patient and thereby provide recommendations for its use.

**Methods**

A systematic search of studies comparing capnography monitoring and non-capnography monitoring in the spontaneously breathing, non-intubated emergency patient, published in the MEDLINE, SCOPUS, Cochrane, Academic Search Complete, and CINAHL databases from 1^st^ of January, 1990 up to 24^th^ of February, 2016 were performed. In total 409 studies were identified of which 11 (one meta-analysis, two randomised controlled trials, eight cohort studies) met pre-specified inclusion criteria, involving 1,450 study subjects.

**Results**

The retrieved literature suggests capnography beneficial to clinical decision making in the investigated population. The included studies were, however, found to be of variable quality and strength with several risks of bias, methodological and technical limitations which led to questionable and inadequate conclusions.

**Conclusions**

This review found some sporadic benefits to clinical decision making when applying capnography to the spontaneously breathing, non-intubated emergency patient. Until more evidence from high quality studies is available, capnography in the investigated cohort should be utilized with a critical approach, as it is not possible to adequately scientifically support recommendations and guidelines for its use.

## A19 Telephone follow-up after discharge does not improve patient satisfaction or readmission rate: a randomized controlled trial

### Mette Elkjær^1^, Christian Jørgensen^1^, Mikkel Brabrand^1,2^, Bibi Gram^2^

#### ^1^Department of Emergency Medicine, Hospital of South West Jutland, Esbjerg, Denmark; ^2^Institute of Regional Health Research, University of Southern Denmark /Centre South West Jutland, Esbjerg, Denmark

##### **Correspondence:** Mette Elkjær (Mette.Elkjaer@rsyd.dk)

**Background**

Insufficient executed discharges from an Emergency Department (ED) may have an impact on the patient experienced satisfaction and increase the risk of readmission. Considerable amounts of information delivered within a short time and transient contacts with various health professionals may lead to uncertainties regarding medicine, symptoms, out-patient visits, follow-up by general practitioner, and rehabilitation. The aim of this study was to investigate if a telephone interview with emphasis on ambiguities regarding discharge may increase patient experienced satisfaction and reduce readmissions.

**Methods**

Patients ≥50 years admitted and discharged directly from an ED were randomly assigned to an intervention group or a control group. The intervention group (I) received a telephone interview 1-3 days following discharge. The control group (C) received standard discharge procedures. Fourteen days after discharge, the patients filled out a questionnaire focusing on individual needs and information on medical side-effects, home care and rehabilitation. The primary outcome was patient experienced satisfaction regarding the discharge estimated on a scale ranging from 1-5 (5 highest). Secondary outcome was number of readmissions. Data will be presented as mean ± SD unless stated.

**Results**

We included 137 patients, 74 in the control group and 63 in the intervention group. Seventy-three were male and 64 female, age 69 ± 10 years, 98 patients were admitted for a medical reason and 49 patients were living alone. Length of stay in the ED was 1401.5 ± 867.8 minutes (range 102 – 5641). At baseline there were no differences in these variables between the groups. Telephone follow-up did not improve the patient experienced satisfaction (I: 4.0 ± 1.2, C: 4.0 ± 1.1, p = 0.93). The intervention did not affect the frequency of readmission with no difference between the groups after the intervention (I: 12 readmissions C: 11 readmissions p = 0.51).

**Conclusion**

A telephone interview following discharge from the ED with emphasis on unsolved questions had no impact on patient experienced satisfaction regarding discharge and did not affect the frequency of readmission. The patients were in general satisfied with the process of discharge and a single phone call was not sufficient to achieve a higher satisfaction.

## A20 Multi-rule out CT-screening of high-risk all-cause patients in an emergency department

### Mia M. Pries-Heje^1^, Rasmus B. Hasselbalch^2^, Lisbet Ravn^3^, Morten N. Lind^3^, Thomas Boel^4^, Peter Sommer Ulriksen^1^

#### ^1^CT Innovation Unit, Department Of Radiology, Herlev Hospital, Herlev, Denmark; ^2^Department Of Cardiology, Herlev Hospital, Herlev, Denmark; ^3^Emergency Department, Herlev Hospital, Herlev, Denmark; ^4^Department Of Gastrointestinal Surgery, Herlev Hospital, Herlev, Denmark

##### **Correspondence:** Mia M. Pries-Heje (miapriesheje@gmail.com)

**Background**

While earlier studies have looked at CT-screening of patients based on symptoms, a screening of patients with CT based on their estimated prognosis alone has not been done before. For moderate-to-high risk patients presenting in the Emergency Department (ED), the potential gain from such a scan might outweigh the risks of the added radiation exposure. Studies show that vital parameters are strong predictors of patient prognosis, and that patients triaged as high-risk (red) or moderate-to-high risk (orange) on vital parameters have a high 30-day mortality rate. Our objective was to investigate the feasibility of an accelerated “multiple-rule-out” CT-screening of moderate-to-high risk patients and its impact on clinical decision making.

**Methods**

Patients triaged as red or orange according to vital parameters, and 40-years or older were eligible for inclusion. ED physicians filled out tentative diagnosis prior to CT scan. Patients were scanned with a combined ECG-gated and dual energy CT-scan of cerebrum, thorax and abdomen. Results and findings of the scan were reported to ED physician by radiologist straight away. The impact of the CT scan on patient diagnosis and treatment was examined prospectively by 2 physicians separately.

**Results**

A total of 100 patients were included in the study, (53% female, median age 73 years [range 43-93]). The triage level according to vital parameters was orange for 2/3 (67%) of patients and the 30-day all-cause mortality was n = 7 (7%). Of the included patients, n = 16 (16%) would have had a CT-scan performed on a clinical indication. The scan lead to change in treatment or additional examinations in n = 38 (38%) of cases, n = 24 (24%) of which were diagnostically significant, change in acute treatment for n = 11 (11%) and previously unrecognized malignant tumors were found in n = 10 (10%) cases. The mean size-specific dose estimate pr. CT-scan for included patients was 16.0 mSv.

**Conclusion**

Screening with a multi-rule out CT scan of high-risk patients in an ED is feasible and result in discovery of clinical unrecognized diagnoses and malignant tumors, but at the cost of higher radiation exposure and additional exams without diagnostic significance.

## A21 Admissions to the emergency department are not always preceded by a visit to a general practitioner: results from a national flash-mob investigation

### Nadia Hejgaard Jensen^1^, Kristian Møller Jensen^1^, Elise Mølleskov^2^, Iben Østergaard Fog^2^, Mette Rahbek Kristensen^3^, Ellen Jensen^4^

#### ^1^University of Southern Denmark, Odense, Denmark; ^2^University of Copenhagen, Copenhagen, Denmark; ^3^Department of Emergency Medicine, Hospital of South West Jutland, Esbjerg, Denmark; ^4^University of Aalborg, Aalborg, Denmark

##### **Correspondence:** Nadia Hejgaard Jensen (Najen10@student.sdu.dk)

**Background**

Danish general practitioners (GP) are gatekeepers to the secondary and tertiary healthcare system, and all Danish emergency department (ED) attendances require prior contact to the healthcare system (either telephone hotline, GP service or ambulance services). Thus, the secondary healthcare system, especially EDs, are based on the assumption that a large proportion of patients are seen and treated by GPs and only admitted to an ED in case of severe or complicated illness. As only little data is available on this, we performed a nationwide cross-sectional survey to investigate how patients are transported to the ED and how many have had contact with a physician prior to arrival.

**Methods**

We simultaneously collected data from six EDs from all five Danish healthcare regions on a Monday in late August from 10 am-10 pm. By interviewing each patient, we recorded gender, age (in 10-year intervals), triage level, assigned specialty, method of transport to the ED and any prior contact with a physician or nurse. Only adult patients speaking Danish and able to give informed consent were included. Data will be presented descriptively.

**Results**

433 patients were asked to participate and 353 could be included. Of these, 48% were between 50 and 80 years old and 49% were women. 116 (36%) arrived by ambulance and 167 (51%) transported themselves to hospital. 119 (37%) had been seen by – and 64 (20%) had had telephone contact with – a GP prior to admission. 60 (19%) had prior telephone contact with a nurse and 73 (23%) had no prior contact with a healthcare provider before arrival at the ED. 83 (60%) of patients with medical problems had prior contact with a physician while 74 (51%) of patients with an orthopedic problem had.

**Conclusion**

Most of the patients that arrive at the emergency department have been in contact with primary healthcare services, but 23% of all admitted patients arrive without prior contact to the primary healthcare system.

